# Paulistine—The Functional Duality of a Wasp Venom Peptide Toxin

**DOI:** 10.3390/toxins8030061

**Published:** 2016-02-29

**Authors:** Helen Andrade Arcuri, Paulo Cesar Gomes, Bibiana Monson de Souza, Nathalia Baptista Dias, Patrícia Brigatte, Rodrigo Guerino Stabeli, Mario Sergio Palma

**Affiliations:** 1 Department of Biology/Center of Study of Social Insects, Institute of Biosciences, São Paulo State University (UNESP), Rio Claro 13506-900, SP, Brazil; helenaarcuri@ibest.com.br (H.A.A.); pccesar@facfar.unesp.br (P.C.G.); bmsousa@rc.unesp.br (B.M.S.); nathdias@rc.unesp.br (N.B.D.); 2 Fundação Oswaldo Cruz—Health Ministry/Fiocruz Rondônia, Porto Velho 21040-900, RO, Brazil; patbtte@usp.br (P.B.); stabeli@unir.br (R.G.S); 3 Department of Clinical Analysis, Proteomic Center, Faculty of Pharmaceutical, Sciences, São Paulo State University (UNESP), Araraquara 14801-902, SP, Brazil

**Keywords:** wasp venom, inflammation, pain, peptide synthesis, molecular modeling, docking

## Abstract

It has been reported that Paulistine in the venom of the wasp *Polybia paulista* co-exists as two different forms: an oxidized form presenting a compact structure due to the presence of a disulfide bridge, which causes inflammation through an apparent interaction with receptors in the 5-lipoxygenase pathway, and a naturally reduced form (without the disulfide bridge) that exists in a linear conformation and which also causes hyperalgesia and acts in the cyclooxygenase type II pathway. The reduced peptide was acetamidomethylated (Acm-Paulistine) to stabilize this form, and it still maintained its typical inflammatory activity. Oxidized Paulistine docks onto PGHS2 (COX-2) molecules, blocking the access of oxygen to the heme group and inhibiting the inflammatory activity of Acm-Paulistine in the cyclooxygenase type II pathway. Docking simulations revealed that the site of the docking of Paulistine within the PGHS2 molecule is unusual among commercial inhibitors of the enzyme, with an affinity potentially much higher than those observed for traditional anti-inflammatory drugs. Therefore, Paulistine causes inflammatory activity at the level of the 5-lipooxygenase pathway and, in parallel, it competes with its reduced form in relation to the activation of the cyclooxygenase pathway. Thus, while the reduced Paulistine causes inflammation, its oxidized form is a potent inhibitor of this activity.

## 1. Introduction

The venoms of the social Hymenoptera insects are part of their chemical arsenal for defensive purposes. Venom components are mostly toxins that interact with different pharmacological targets and cause pain, inflammation, tachycardia/bradycardia, cardiac arrhythmia, and sometimes neurotoxicity [1,2,3,4].

Wasp venoms contain a complex mixture of different types of compounds such as proteins, peptides (which represent up to 70% of the dry content), amino acids, and biogenic amines [5]. Most of these peptides are linear, polycationic and amphipathic molecules. The identification and pharmacological characterization of the peptides in venoms are important steps in understanding the symptoms observed during the envenoming process. In social wasp venoms, peptide toxins are generally classified into families according to the type of biological activities they exhibit: (i) mastoparans cause mast cells degranulation; (ii) chemotactic peptides promote chemotaxis of polymorphonucleated leukocytes [6]; (iii) wasp kinins are known to cause pain; (iv) very short linear peptides are known for their chemotactic and inflammatory activities; and (v) a novel class of peptides presenting an intramolecular disulfide bridge presents different profiles of inflammatory activities [7,8,9].

**Figure 1:**
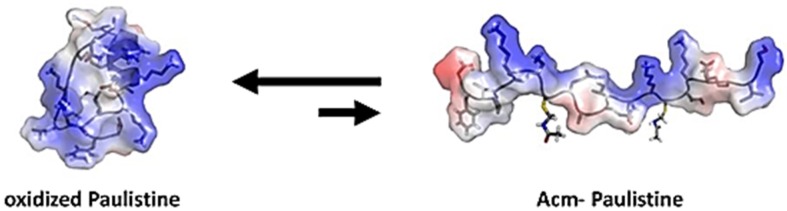
The structural models of oxidized Paulistine and reduced and acetamidomethylated Paulistine under equilibrium as surface charge (electrostatic) representations. The negative residues are shown in red, and the positive residues are highlighted in blue.

A novel peptide belonging to the last class of wasp toxins was recently reported in the venom of the wasp *Polybia paulista*. It is known as Paulistine and has 21 amino acid residues in its sequence, with a single intramolecular disulfide bridge. It was demonstrated that the peptide co-exists in two different forms (Figure 1): (i) one naturally oxidized form, which is more abundant, presents a more compact structure because of the presence of a disulfide bridge; and (ii) the second form, which is naturally reduced, *i.e.*, without the disulfide bridge, exists in a linear (expanded) conformation. The use of assays of pain and inflammation in presence and absence of a series of generic and selective inhibitors for the 5-LO and for the cyclooxygenases-1 and -2, permitted the identification of the exact molecular target of each form of Paulistine (oxidized/reduced) [10]. Thus, it was reported that the oxidized Paulistine causes hyperalgesia, due to an apparent interaction with receptors involved in mediation of lipid metabolism at level of the 5-lipoxygenase (5-LO) pathway; meanwhile, the reduced Paulistine causes hyperalgesia, and seems to act on the receptors involved in prostanoid metabolism, more specifically, the cyclooxygenase type II pathway [10].

**Figure 2:**
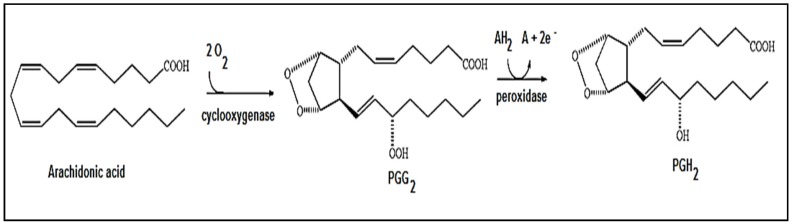
The simplified reaction scheme for the conversion of arachidonic acid into prostaglandin G2 at the cyclooxygenase site, and in turn, the conversion of this compound into prostaglandin H2 at the peroxidase site by the enzyme PGHS2.

The enzyme cyclooxygenase-2 (COX-2), also known as prostaglandin-endoperoxide synthase 2 (PGHS2), is involved in the conversion of arachidonic acid to prostaglandin H2 [11,12]. Structurally, PGHS2 is a homodimer, and its subunits have three different structural domains: (i) a short *N*-terminal epidermal growth factor (EGF) domain; (ii) an α-helical membrane-binding moiety; and (iii) a *C*-terminal catalytic domain. Both monomers present two physically distinct catalytic sites: a cyclooxygenase site and a peroxidase site [12]. This enzyme catalyzes the conversion of arachidonic acid to prostaglandins in a two-step reaction: (i) hydrogen is extracted from arachidonic acid, and then two molecules of oxygen are added by the enzyme to arachidonic acid, resulting in the formation of PGG2 by the cyclooxygenase site; and (ii) then, PGG2 is converted to PGH2 by the peroxidase site [13] (Figure 2). PGHS2 is then converted into a series of other prostaglandins (Figure 2).

Despite evidence that it is a homodimer, it was reported that PGHS2 functions as a conformational heterodimer, *i.e.*, one of the monomers is considered the catalytic one, while the other one functions as an allosteric sub-unit [14]. The heme prosthetic group binds only to the peroxidase site. Meanwhile, arachidonic acid binds to the cyclooxygenase site of the catalytic sub-unit. The substrate can also bind to the allosteric subunit with an affinity much higher than that observed for the catalytic subunit, but this only induces conformational changes in this monomer for the allosteric regulation of the catalytic subunit [12,15]. The binding of non-substrate fatty acids can potentiate/attenuate the effects of PGHS2 inhibitors, depending on the type of fatty acids and whether the inhibitor binds to the catalytic or allosteric subunit [15].

**Figure 3:**
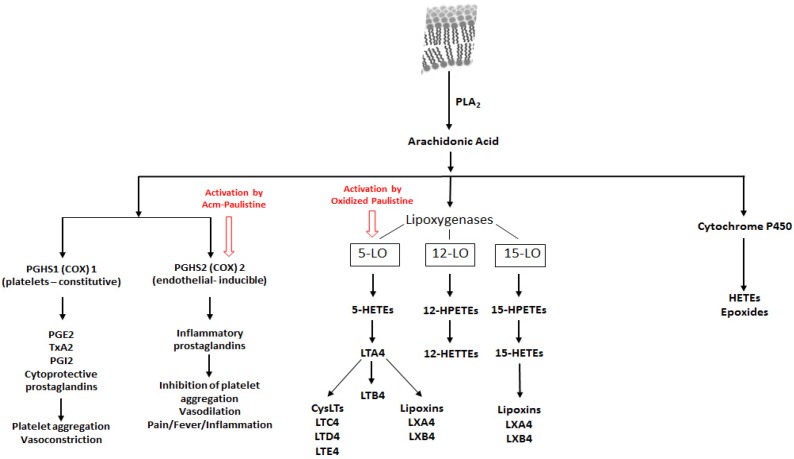
A schematized chart showing the metabolic pathways of arachidonic acid, focusing mainly on the cyclooxygenase and the lipooxygenases routes. The red arrows indicate the sites of actions of Acm-Paulistine and Paulistine. Phospholipase A2 (PLA2); prostaglandin-endoperoxide synthase (PGHS); cyclooxygenase (COX); lipoxygenase (LO); Prostaglandin E2 (PGE2); tromboxane A2 (TxA2); prostaglandin I2 (PGI2); hydroxyeicosatetraenoic acid (HETE); peroxyeicosatetraenoic acids (PETE); cys-leukotriene (CysLT); leukotriene C4 (LTC4); leukotriene D4 (LTD4); leukotriene (E4 LTE4); lipoxin A4 (LXA4); and, lipoxin B4 (LXB4).

As schematized in Figure 3, PGHS2 occurs into two forms: (i) a constitutive form, PGHS1; and (ii) an inducible form, PGHS2, which is expressed in several cell types in response to growth factors, and cytokines, at the site of inflammation [16]. The cyclooxygenases catalyze the first step in the synthesis of prostanoids, a large family of metabolites of arachidonic acid, such as prostaglandins, prostacyclin, and thromboxanes [17,18]. Prostaglandins are important mediators of physiological functions like vasodilatation, gastric cytoprotection, renal homeostasis, and platelet aggregation; this class of compounds also plays a major role in mediating fever, pain sensitivity, and inflammation [17] (Figure 3). Prostacyclin is a lipid member of the eicosanoid family, which inhibits platelet activation, being also an effective vasodilator [19]. Thromboxanes are eicosanoid compounds acting as vasoconstrictors, being potent hypertensive agents; they also activates platelet aggregation, promoting the formation of blood cloths, and reducing the blood flow around the site of clothing [20]. PGHS2 is one of the major components in inflammatory reactions in peripheral tissues [17]. The induction and overexpression of PGHS2 has been associated to increased levels of prostaglandin E2 (PGE2), which in turn seems to modulate mechanisms of cell proliferation/cell death, and even tumor invasion in different cancer types such lung, colon, and breast [18]; elevated levels of expression of PGHS2 are also associated to diseases related to chronic inflammation such Crohn’s disease, and osteoarthritis [21,22]. PGHS2 has attracted the attention of the pharmaceutical companies for the development of selective inhibitors, which could be used both as anti-inflammatory compounds, as well to inhibit the proliferation of different types of cancer cells [17,23,24].

Considering that the reduced form of Paulistine may spontaneously oxidize to form the disulfide bridge, we reduced and acetamidomethyled the peptide *in vitro* to stabilize its linear conformation (Acm-Paulistine). The *in vitro*-modified peptide maintained its activity in relation to the cyclooxygenase type II pathway [10].

Taking into account that Paulistine is a toxin, it is comprehensive in that it exists in the venom in two different conformations, with each conformation individually active against a different type of inflammatory receptor. This structural feature enlarges the number of molecular targets for the same toxin. Thus, Paulistine and Acm-Paulistine are considered interesting tools to investigate the mechanisms of pain and inflammation during wasp venom envenoming. In the present study, we decided to investigate the mechanism of Paulistine as an inhibitor of the inflammatory process involving the cyclooxygenase type II pathway. A simulation of the docking of both forms of the peptide with PGHS2 revealed an unusual interaction between the enzyme and the oxidized form of Paulistine, suggesting the potential use of this peptide as a model for the rational development of a novel drug to be used as an analgesic and/or an anti-inflammatory drug for processes caused by the activation of the cyclooxygenase type II pathway.

## 2. Results and Discussion

**Figure 4:**
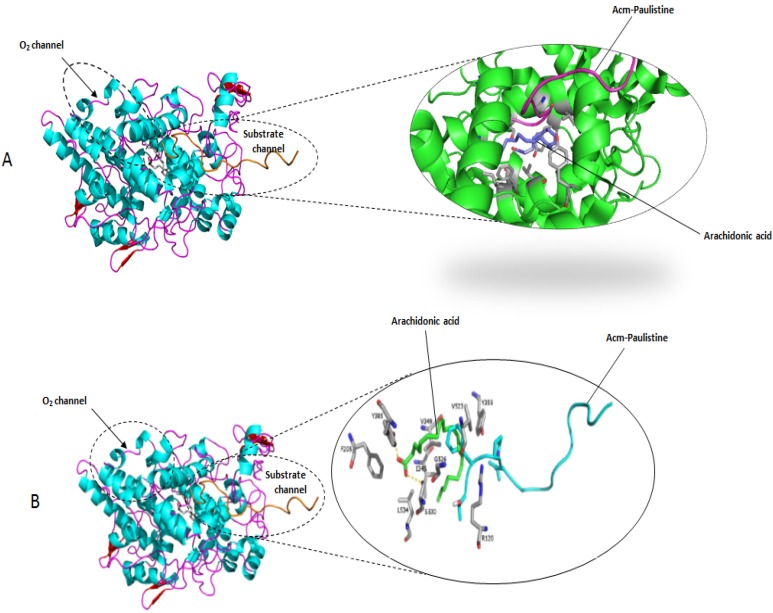
(**A**) The molecular models of the peptide Acm-Paulistine (pink) and arachidonic acid (purple) docked at their binding site in the substrate channel of catalytic subunit of PGHS2 represented by ribbon diagrams. (**B**) A detailed representation of the binding site of Acm-Paulistine with the main amino acid residues involved in the interaction of the peptide with the docking site: R120, F205, I345, Y355, V349, Y385, V523, G526, S530, and L534. The molecular model of the allosteric sub-unit was not shown in this figure in order to simplify it.

**Figure 5:**
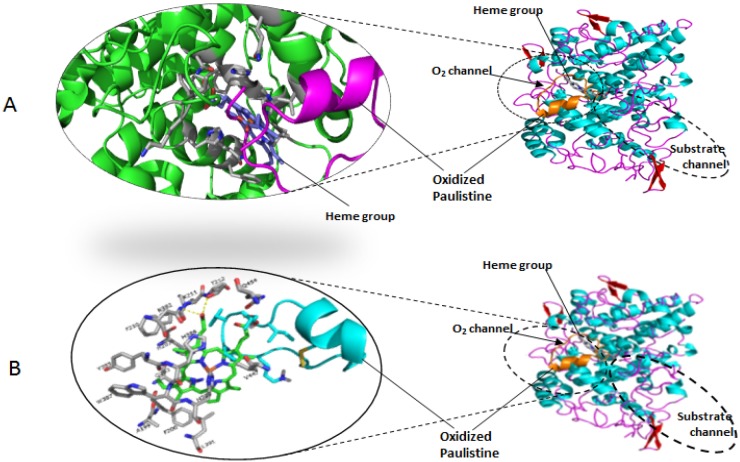
(**A**) The molecular models of the peptide Paulistine (pink) docked at its binding site in the O_2_ channel of the catalytic subunit of PGHS2 (green) represented by ribbon diagrams. The heme group is also represented in this figure (purple). (**B**) A detailed representation of the binding site of Paulistine showing the main amino acid residues involved in the interaction of the peptide with the docking site: W387, A199, F200, L391, Y385, Q203, H388, F210, H207, H386, V447, K211, T212, and Q454. The molecular model of the allosteric sub-unit was not shown in this figure in order to simplify it.

To identify the possible nature of the interactions between Paulistine and Acm-Paulistine with PGHS2, the molecular docking between the enzyme and both forms of the peptide was simulated (Figure 4 and Figure 5, respectively). For this purpose, the molecular model of murine PGHS2 (PDB code: 3LN1) without any ligand was used. A murine enzyme was selected because the assays of pain and inflammation developed in the present study were experimentally performed with a rat model. All the docking simulations were performed with the complete structure of PGHS2 (catalytic and allosteric unit); however, the figures of the molecular models all over the manuscript are showing only the catalytic sub-unit, to make easy the visualization of the structural details.

The interaction with the ligands revealed that the molecule of arachidonic acid forms H-bonds with the residues Tyr385 and Ile345 of the catalytic sub-unit, while Acm-Paulistine is positioned at the entrance of the substrate channel between the residues Tyr355 and Arg120 (Figure 5), favoring the formation of the H-bonds mentioned above at the cyclooxygenase site. Another molecule of the substrate binds to the non-catalytic conformation, in which its carboxylate group interacts with the residues Tyr385 and Ser530 [25]. Meanwhile, the binding of Paulistine occurs in an unusual mode, *i.e.*, within the O_2_ channel between the residues Gln454 and Val447, blocking the access of O_2_ to the heme group within the peroxidase site (Figure 4).

**Figure 6:**
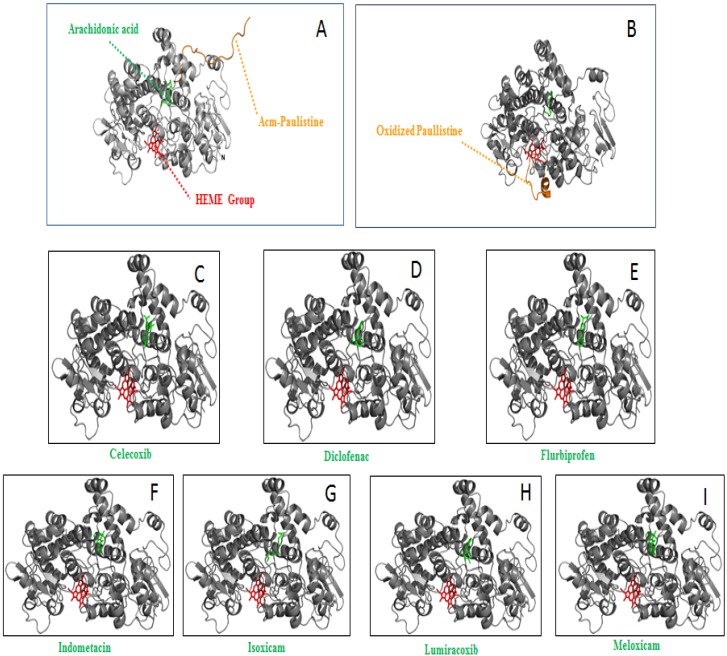
Representations of the dockings of the peptides and a series of inhibitors (anti-inflammatory compounds) in the 3-D structure of the catalytic subunit of PGHS2: (**A**) Acm-Paulistine (orange) and arachidonic acid (green); (**B**) Paulistine (orange) and arachidonic acid (green); (**C**) Celecoxib (green); (**D**) Diclofenac (green); (**E**) Flubiprofen (green); (**F**) Indometacin (green); (**G**) Isoxican (green); (**H**) Lumiracoxib (green); and (**I**) Meloxican (green). The cartoon also includes the positioning of the heme group (red). The molecular model of the allosteric sub-unit was not shown in each one of these figures in order to simplify them.

To compare the interactions of Paulistine, Acm-Paulistine, and some classical anti-inflammatory drugs with PGHS2, ligands docking with the enzyme were simulated; thus, the compounds Indometacin, Diclofenac, Flurbiprofen, Isoxican, Meloxican, Celecoxib, and Lumiracoxib were virtually docked with the enzyme. The results (Figure 6A–I) indicate that all inhibitors bind to the substrate channel close to the binding site of arachidonic acid, as described further down in this paragraph. Classic Non-Steroidal Anti-Inflammatory Drugs (NSAIDs), such as indomethacin, bind through ionic interactions to the side chain of the residue Arg120 within the cyclooxygenase channel, as well through hydrogen bonding with the side chain of the residue Tyr355. These residues are located at the base of the active site, comprising part of the gate connecting the active site and the ligand access channel [14,15,26]. In contrast, the inhibitor Diclofenac makes hydrogen bonds through its carboxylate with the catalytic Tyr385 residue, as well as with Ser530 at the apex of the active site [27]. Another binding mode of the inhibitors is exhibited by diarylheterocycles (Celecoxib and Lumiracoxib), which bind to a side pocket present in the cyclooxygenase channel of PGHS2, but is absent in PGHS1 (COX-1) [15,28,29]. The inhibitors of Oxicam family bind to the active site of PGHS2 through two highly coordinated water molecules. The 4-hydroxyl group on the thiazine ring interacts with residue Ser530 through a hydrogen bond, while the heteroatom of the carboxamide ring interacts with Tyr385 and Ser530 through a highly coordinated water molecule. The nitrogen atom of the thiazine and the oxygen atom of the carboxamide bind to Arg120 and Tyr355 through water molecules [30].

**Figure 7:**
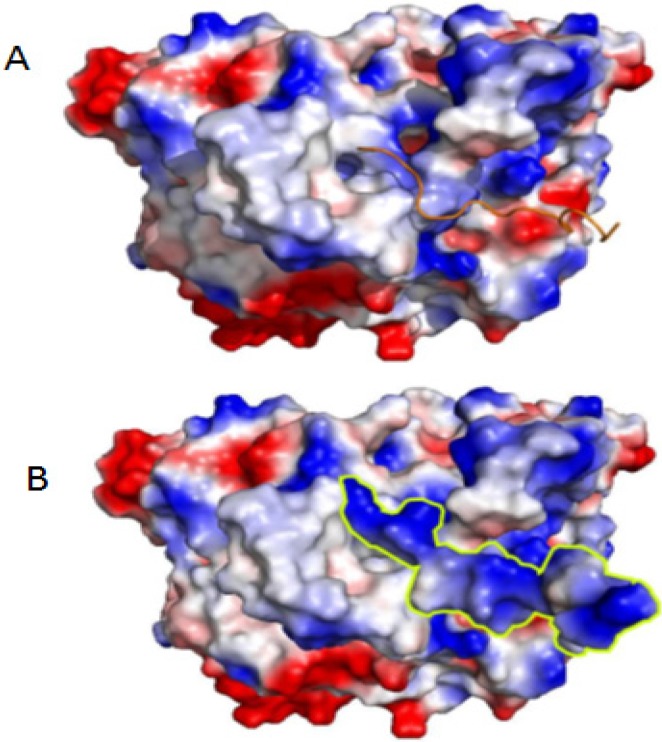
(**A**) Representation of the peptide Paulistine (brown) docked on its binding site on the O_2_ channel of the enzyme PGHS2 (COX-2), represented as electrostatic surface. (**B**) The same representation showing both molecules as electrostatic surface, in which it is possible to observe the charge complementarity of both surfaces. The position of Paulistine is surrounded by a green line. The residues in white represent the neutral ones, while the blue and red regions correspond to the positive and negative regions, respectively.

Using the differential physicochemical parameters of the energy between the final and initial conformations of PGHS2, it was possible to estimate the variation of the free energy for the binding (ΔGbind) of the ligands (Paulistine and anti-inflammatory compounds) in the enzyme, as shown in Table 1. This table reveals that the peptide Paulistine and the inhibitors Indometacin, Diclofenac, Isoxican, Meloxican, and Lumiracoxib have negative values for ΔGbind, suggesting that these compounds can interact spontaneously with PGHS2 with high affinity. Figure 7 shows that there is charge and van der Waals complementarity between the peptide Paulistine and the enzyme PGHS2. A comparison between the values of free energy of formation (Table 1), solvation energy, and hydrogen bonding formation for the complex protein:peptide obtained from docking simulations and PISA, revealed very similar values determined by both softwares (not shown results). The values of surface area and physicochemical properties (hydrophocity and electrostatic surface distribution) were calculated with PISA and confirmed by using PYMOL.

The commercial inhibitors of PGHS2 generally bind to the pocket of access used by the substrate to enter its binding site, such as that mentioned for Celecoxib [12]. It is unknown whether the commercial anti-inflammatory drugs block the access of oxygen to the heme group, as observed for the binding of Paulistine to PGHS2 (Figure 4). The results of the docking simulations suggest the possibility that the hyperalgesic effect caused by Acm-Paulistine may be, at least in part, reverted by the binding of Paulistine to PGHS2. Therefore, the results of Table 1 suggest that Paulistine could bind to PGHS2 with a higher affinity than the classical inhibitors.

**Figure 8:**
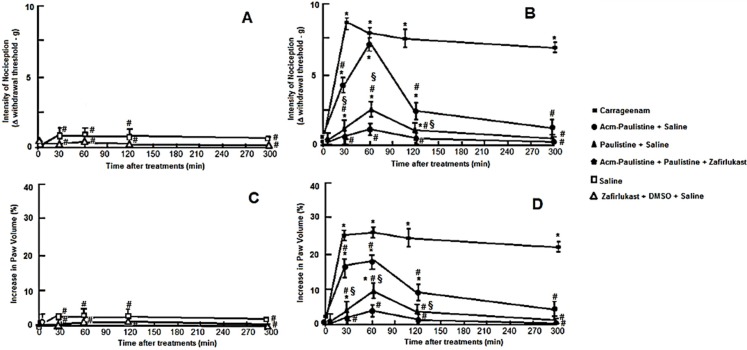
Evaluation of the hyperalgesic and edematogenic effects of Paulistine and Acm-Paulistine. The paw threshold was estimated using an electronic von Frey device. The force needed to induce paw withdrawal was recorded as the pain threshold represented by delta (Δ) (**A**,**B**). Edematogenic effects were evaluated using a digital caliper pachymeter (**C**,**D**). The data were obtained at times 0, 30, 60, 120 and 300 min after peptides administration (10 μg i.pl. shown in **A**–**D**), or Carrageenan administration (Cg, 300 μg i.pl.), or Zafirlukast (5 mg/Kg by oral route) in 0.5% DMSO administration. The negative control groups (**A**,**C**) consisted of: (i) animals injected with sterile saline; and (ii) animals treated with Zafirlukast in DMSO and sterile saline. The group considered as the positive control (**B**,**D**) consisted of animals treated with Carrageenam. The results are expressed as the mean ± SEM of five animals per group. * *p* < 0.001, a significant difference compared with the mean values of the saline group; § *p* < 0.001, a significant difference compared with the mean values of the “Zafirlukast + DMSO + saline” group; and # *p* < 0.001, a significant difference compared with the mean values of the carrageenan group.

To verify the potential binding of Paulistine to PGHS2, inhibiting the inflammatory action caused by Acm-Paulistine, the involvement of both forms of Paulistine in the induction of inflammation and pain processes through the cyclooxygenase II pathway were investigated. For these purposes, 10 μg of each peptide alone, or both peptides together were injected into mice by intraplantar (i.pl.) administration, and the mice were tested for mechanical hyperalgesia (through the electronic von Frey) and edema formation (through the use of a digital paquimeter) at different times (30, 60, 120, and 300 min) after the peptide administration. The results of these assays are shown in Figure 8A–D. The animals of control groups were injected with saline or treated with Zafirlukast (5 mg/Kg by oral route) in 0.5% DMSO prepared in sterile saline solution. The results shown that 60 min after the injection of the Acm-Paulistine there was a significant peak of pain (similar to that caused by the carrageenan); after this time, a decrease in pain threshold was observed, which was characterized as hypernociception (hyperalgesia) (Figure 8B). This hyperalgesia decreased until it almost disappeared 300 min after peptide administration. The animals treated with carrageenan (positive control) exhibited hyperalgesia until the end of the observations (300 min) (Figure 8B). No significant differences were observed in the nociceptive threshold of the control groups (Figure 8A). The results got for the oxidized Paulistine presented a general profile similar to that presented by Acm-Paulistine, except that the intensity of the nociception was much more reduced than that observed for the reduced peptide (Figure 8B). This assay was performed considering that the oxidized form of Paulistine could inhibit the inflammation and pain induced by Acm-Paulistine acting on the receptors of the cyclooxygenase pathway II (Figure 2) when both peptides were assayed together. To inhibit the action of oxidized Paulistine on the 5-LO pathway, Zafirlukast, a leukotriene receptor antagonist, was included in this assay. The 5-LO pathway antagonist was administrated prior to peptide injection. The administration of both peptides together revealed that oxidized Paulistine is able to inhibit the hyperalgesia caused by Acm-Paulistine (Figure 8A,B).

The action of both peptides in relation to the formation of edema was also investigated (Figure 8C,D). The results showed that 10 μg of the Acm-Paulistine induced a significant increase in paw thickness up to 60 min after the peptide injection; the edema decreased continuously up to 300 min (Figure 8D). The paw thickness induced by the oxidized Paulistine was lower than that induced by Acm-Paulistine; the activity of both peptides were much more reduced that that presented by the positive control (Carrageenam) (Figure 8D). The administration of both peptides together revealed that oxidized Paulistine is able to inhibit the edema formation caused by Acm-Paulistine (Figure 8B); the combined use of Zafirlukast prior to peptide injection, ensured that apparently there is no contribution of 5-LO in this process. Therefore, it seems that the results above represent more evidence for the hypothesis raised above (based on docking simulations); it predicts that oxidized Paulisitne may dock at the O_2_ channel of PGHS2, blocking the access of O_2_ to the prosthetic heme group within the peroxidase site, inhibiting the formation of the mediators of inflammation and pain produced by the cyclooxygenase pathway.

The existence of the same peptide toxin existing in two different forms, with different selectivities for activating different inflammatory processes, is beneficial for the social wasp, because this structural and functional features enlarges the number of molecular targets of the same toxin during envenoming caused by *P. paulista.*

The unusual binding of Paulistine to the PGHS2 molecule, blocking the access of oxygen to the prosthetic heme group, inhibits the cyclooxygenase activity. Under the toxinological point of view, this inhibition would favor the inflammatory process induced by the activation of the 5-LO pathway during the envenoming process. The mechanism of the envenoming caused by stinging of social Hymenoptera against their predators is based on mnemonic actions, *i.e.*, the envenoming results in no lethality or apparent morbidity, but causes physical discomfort promoted by a series of pharmacological actions. The physical discomfort generally becomes associated with the presence of the wasps, which is enough to remind the predators of unpleasant previous experiences with these insects, keeping predators (or intruders) far away from their colonies [31]. The activation of the 5-LO pathway results mainly in the production of leukotrienes, which in turn may trigger the contraction of smooth muscle in the bronchioles and also may cause asthma and rhinitis [32]. Meanwhile, the activation of the cyclooxygenases type II pathway generally results in the production of prostaglandins, which in turn causes inflammation and pain around the site of venom inoculation and sometimes fever [33]. Thus, the fostering of the activation of the 5-LO pathway seems to cause more extensive effects, and thus be more suitable for mnemonic purposes [34].

Under the bioprospective point of view of the binding of Paulistine to the pocket of oxygen access to the heme group of PGHS2 represents an interesting subject of investigation for the future, because it may contribute to the design of novel anti-inflammatory drugs. NSAIDs are widely used and may cause a series of side effects, depending on the dosage and the type of use/administration. Therefore, compounds that might become models for the rational development of novel anti-inflammatory drugs are continuously demanded by pharmaceutical companies [29,30]. Considering the uncommon type of inhibition of PGHS2 promoted by Paulistine, this peptide has potential to be exploited in the future as a model for the rational development of selective PGHS2 (COX-2) inhibitors.

## 3. Experimental Section

### 3.1. Peptide Synthesis

The peptides were prepared by using stepwise solid-phase synthesis based on the chemistry of *N*-9-fluorophenylmethoxy-carbonyl (Fmoc) with the resin NovaSyn TGS resin (NOVABIOCHEM). The protective groups used for the side-chains were β-t-butyl ester (OtBu) for aspartic acid, t-butyl (tBu) for threonine and serine, trityl (Trt) for glutamine, and t-butoxycarbonyl (Boc) for lysine. The side-chain protective group Trt was used in the synthesis of the oxidized peptide (with the disulfide bridge); the permanent blocking of free thiol groups of cysteine residues was performed by acetamidemethylation (Acm).

The mixture trifluoroacetic acid/1,2-ethanedithiol/anisole/phenol/water (82.5:2.5:5:5:5 by volume) was used for the cleavage of peptide-resin at room temperature for 2 h. The material of synthesis was filtered for removal of the resin, and then added cold (4 °C) anhydrous diethyl ether (Sigma) to precipitate crude peptide, which was collected as sediment after centrifugation at 1000 g at 15 min. The crude peptide was solubilized in water and purified by RP-HPLC. The peptide with the disulfide bridge was obtained by the air oxidation of cysteine residues as previously described [9].

Synthetic Paulistine peptides, including the oxidized form and the reduced and acetoamidated forms, were purified by HPLC (mod. LC 8A, Shimadzu, Kyoto, Japan) using a C18 column (30 mm × 250 mm, 15 µm) (Shim-Pack ODS Prep, Shimadzu, Kyoto, Japan), with an isocratic elution of 45% (*v*/*v*) MeCN/H2O (containing 0.05% (*v*/*v*) TFA) at a flow rate of 10 mL/min. The elution was monitored at 214 nm with a UV-Vis detector (Shimadzu, mod. SPD-20A Prominence, Kyoto, Japan). The fractions were collected in glass vials, and latter dried in a lyophilizer (Heto, Denmark). The homogeneity and the accuracy of the sequence of the synthetic peptides were assessed by the gas-phase sequencer PPSQ-21A (Shimadzu, Kyoto, Japan) using automated Edman degradation chemistry and ESI-MS analysis.

### 3.2. Mass Spectrometry

The mass spectrometric analyses were performed using an ion trap-time of flight hybrid mass spectrometer (LCMS-IT-TOF) produced by Shimadzu Co. (Shimadzu, Kyoto, Japan) The samples were injected into the electrospray transport solvent with an autosampler injector at a flow rate of 0.2 mL/min of MeCN 50% (*v*/*v*). Mass spectrometric analyses were carried-out using positive electrospray ionization (ESI+) under the conditions described bellow: CDL temperature was adjusted to 200 °C, 4.5 kV for the voltage of capillaries, 3.5 V was set for the cone lens, a flow rate of the nebulizer gas (nitrogen) of approximately 1.5 L/h and a drying gas (nitrogen) at a flow rate of 100 L/h. Mass spectra were continuously acquired in the range of *m*/*z* 50 to *m*/*z* 4000, in a time window of 2 min. The data were acquired and analyzed using LCMS Solution software (Shimadzu, Kyoto, Japan).

### 3.3. Molecular Modeling

The search for templates of the sequence of Paulistine was performed using the BLASTP tool and the alignment was formatted and input into the program. To solve the structure of Paulistine, a template was selected at Protein Data Bank (PDB) using the experimental data of a homologous peptide solved by NMR (PDB ID: 2KGH). Synthetic peptide models were built using restraint-based modeling implemented in MODELLER (Version 9.16, San Francisco, CA, USA, 2016) [35] with standard protocols for the comparative protein structure modeling by satisfying the spatial restrains [36]. For each peptide, a total of 1000 models were created, and the best models were selected using the MODELLER objective function [36] and the stereo chemical analysis of PROCHECK [37]. The sequence similarity between Paulistine and the template was 61% (with a sequence identity of 38%). The final models were selected. The models selected had 100% of their residues in the favored regions of the Ramachandran plot, with the best values of the overall G-factor, and the models had lower values of energy minimization. Both reduced and oxidized forms of Paulistine were modeled. The reduced peptide was modeled considering the acetamidomethylation of the thiol groups of cysteine. Pymol was used for the visualization of the models of both forms of Paulistine [38]. After molecular modeling, both peptides were submitted to molecular dynamics simulations in order to study the conformational changes of these peptides as published elsewhere [10].

### 3.4. Molecular Docking Simulations

One of the fundamental questions in structural biology is related to the study of the interaction between a protein and a ligand, and an effective method used to study this interaction is the molecular docking simulation. This type of study is very important for pharmaceutical applications, such as structure-based drug design, because these ligands can inhibit vital proteins.

The molecular docking of ligand agonists to the PGHS2 structure was carried out using the AutoDock3.0.5 software package [39]. All the torsion angles in the small molecules were set free to perform flexible docking. Polar hydrogen atoms were added to PGHS2 by using the hydrogen module in AutoDock Tools (ADT), and the empirical free energy function and the Lamarckian genetic algorithm (LGA) were used for docking. The atomic coordinates of the ligands were obtained from the PDB.

The molecular docking of the structure of PGHS2 with both forms of Paulistine peptide was carried out using the PatchDock web server, version 1.3 [40]. The atomic coordinates of the complete protein (dimer) without its ligands were obtained from the PDB (access code 3LN1) [41]. A global search for all possible binding configurations of the protein and each peptide were made, and a total of 1000 predictions were obtained using PatchDock, which was also used to calculate the biding energy. The ten best models were chosen, refined and ranked again with FireDock.

PDBe PISA, which is an interactive tool for the exploration of macromolecular interfaces, was used to analyze the surface area, physicochemical properties, and to predict free energy of formation, the solvation energy gain and the hydrogen bonds across the interface of the protein:peptide complex after molecular docking simulation [42].

### 3.5. Biological Assays

#### 3.5.1. Hyperalgesic and Edematogenic Effects

Male Swiss mice weighing between 25 and 30 g were used throughout this study. The housing of mice occurred under controlled humidity (65% ± 5%) and temperature (22 °C ± 1), in a sound-attenuated room subjected to a 12 h light–dark cycle. Food and water were available *ad libitum*, and mice were taken to the testing room at least 1 h before the experiment. All behavioral testing was performed between 9:00 a.m. and 4:00 p.m. The mice were used only once. All experiments were completed in accordance with the Guidelines for the Ethical Use of Conscious Animals in Pain Research published by the International Association for the Study of Pain [43] and the EC Directive 86/609/EEC for Animal Experiments. The animal manipulation protocols were approved by the Institutional Animals Care Committee (CEUA-IB, protocol 016/2011).

#### 3.5.2. Von Frey Electronic Pressure Meter Paw Tests for Mice

Mice were placed in appropriated acrylic cages containing wired gridding floor 30 min before the assay. During this adaptation period, the paws of the mice were poked 2–3 times. Before paw stimulation, the animals remained quiet and presenting neither exploratory movements nor resting on their paws. In these experiments, it was used an electronic von Frey anesthesiometer, a paw pressure meter fitted with a 0.5 mm^2^ plastic tip (IITC Inc., Life Science Instruments, Woodland Hills, CA, USA).

A mirror fit below the gridding floor provided a view of the hind paw movements. The use of an electronic von Frey instrument (Insight, Ribeirão Prêto, SP, Brazil) permitted the automatic register of force intensity of the stimulus by the time of paw withdrawn. The maximal force applied was 18 g. The stimulations were repeated until the mice presented at least two similar measurements. If the results were inconsistent (*i.e.*, a great difference in the baseline response compared to the other animals of the experiment), another animal was used. The hyperalgesic effect was induced by the injection through intraplantar (i.pl.) route into one of the hind paws, of either carrageenan (300 μg) or each form of Paulistine (10 μg). The results are reported as the Δ (delta) withdrawal threshold (g).

#### 3.5.3. Evaluation of Edema

Edema was induced by the injection of carrageenan into one of the hind paws by the intraplantar (i.pl.) route. The increase of the volume of paws (edema) at the region of tibio-tarsal articulation was measured with a digital paquimeter (São Paulo, SP, Brazil). Indomethacin and Zileuton were administered to evaluate their anti-edematogenic effects. The difference between the values measured in each paw were expressed as percent increase of paw volume.

#### 3.5.4. Evaluation of Nociceptivity

For evaluation of the nociceptive activity of Paulistine and Acm-Paulistine, both peptides were dissolved in sterile saline (10 μg/50 μL saline) and applied by i.pl. route in one of the hind paws of the mice. The tip of the pressure meter was introduced in the central area of the hind paw (where the compounds were applied with the help of a 26-G needle) to measure the intensity and the time of paw withdrawn. Nociceptivity was evaluated at 30, 60, 120 and 300 min after Paulistine and Acm-Paulistine application) and compared to the control. Carrageenan (Marine Colloids, 300 μg/50 μL sterile saline) was used as the positive control, while the negative control was performed with sterile saline. To evaluate the involvement of leukotriene receptors, Zafirlukast (SIGMA, Saint Louis, MO, USA) was diluted in 0.5% dimethyl sulfoxide (DMSO, SIGMA, SIGMA, Saint Louis, MO, USA) and administered by an oral route at 5.0 mg/k; animals treated with 0.5% (*v*/*v*) DMSO solution in sterile saline were used as the control group. The detailed involvement of the different pathways of arachidonic acid metabolism in this process was assayed as previously reported [10], with the use of indomethacin (a cyclooxygenase inhibitor), Celecoxib (a type 2 cyclooxygenase inhibitor), and Zileuton (a lipoxygenase inhibitor).

### 3.6. Statistical Analysis

A two-way analysis variance (ANOVA) was used to compare the groups and doses over all times. Three factors were analyzed: the treatments, the time and the time *vs.* treatment interaction. When a significant time *vs.* treatment interaction was detected, a one-way ANOVA followed by Tukey’s test was performed for each time point to distinguish the dose effects [44]. The results with *p* < 0.05 were considered to be significant.

## Figures and Tables

**Table 1:** Values of ∆G_bind_ and T∆S calculated for the oxidized form of Paulistine and a series of commercial inhibitors of COX-2 (anti-inflammatory drugs) *.

Ligand	Bound Surface (Å^2^)	ΔG_bind_ (kcal/mol) *	TΔS (kcal/mol) *
Oxidized Paulistine	1764	−16.5	9.6
Arachidonic acid	940	2.0	4.5
Celecoxibe	1909	2.2	4.8
Diclofenaco	1816	−8.4	3.9
Flurbiprofeno	1739	4.1	3.6
Indometacin	1957	−7.7	4.6
Isoxicam	1842	−6.8	4.2
Lumiracoxibe	692	−11.8	4.0
Meloxicam	1875	−9.4	4.3

* Negative values of ΔG*bind* and positive values of TΔS indicate spontaneous ligand—receptor interaction.
